# Update of antivenom supply for redback spider bites in Japan

**DOI:** 10.1186/s40560-014-0070-3

**Published:** 2015-02-11

**Authors:** Toru Hifumi, Hisashi Taki, Akihiko Yamamoto, Manabu Ato, Yuichi Koido, Yasuhiro Kuroda

**Affiliations:** Emergency Medical Center, Kagawa University Hospital, 1750-1 Ikenobe, Miki, Kita, Kagawa 761-0793 Japan; Ministry of Health, Labour and Welfare, 1-2-2 Kasumigaseki Chiyoda-ku, Tokyo, 100-8916 Japan; Department of Bacteriology II, National Institute of Infectious Disease, Gakuen 4-7-1, Musashimurayama-shi, Tokyo, 208-0011 Japan; Department of Immunology, National Institute of Infectious Disease, Toyama 1-23-1, Shinjuku-ku, Tokyo, 162-8640 Japan; Division of Critical Care Medicine and Trauma, National Hospital Organization Disaster Medical Center, 3256 Midori-cho, Tachikawa, Tokyo, 190-0014 Japan

**Keywords:** Redback spiders, Antivenom, Domestic production

## Abstract

In autumn 2014, with great effort by the Ministry of Health, Labour and Welfare, the research group will obtain several vials of redback spider (RBS) antivenom for emergency use. However, these small amounts of antivenom are insufficient to cover the demands from majority of hospitals in Japan. The research group carefully discussed the domestic RBS antivenom production by themselves for this emergency. We have now entered the second stage for large-scale antivenom production. Although the domestic production of RBS antivenom has started, great caution is required as we move forward with this plan.

## Correspondence

### Letter to the editor

We previously reported that symptoms of redback spider (RBS) bites are usually mild and localized, such as local pain and erythema [1]. However, fatal cases had been reported before the development of antivenom, which is manufactured by the immunization of horses [2,3].

RBSs were found in Metropolitan Tokyo on September 25, 2014, and they are rapidly becoming a nationwide problem in Japan [4]. The definitive treatment for RBS envenomation is to use the specific RBS antivenom produced by the Commonwealth Serum Laboratories (CSL) in Australia. However, a serious issue with the current practice is that RBS antivenom is used as an off-label drug in Japan and must be privately imported from Australia [1].

To compound this issue, RBS antivenom imports from CSL were suspended in autumn 2013. The Ministry of Health, Labour and Welfare (MHLW) launched a research group to evaluate the safety and efficacy of the antivenom and to organize and maintain information on RBS bites from April 2013 [1]. In autumn 2014, with great effort by MHLW, the research group will obtain several vials of antivenom for emergency use. However, these small amounts of antivenom are insufficient to cover the demands from majority of hospitals in Japan.

The research group carefully discussed the option for domestic RBS antivenom production by themselves for this emergency. The first stage started in April 2014. Over 5,000 RBSs were collected, and their venom was extracted by research group in the summer of 2014. We have now entered the second stage of development to evaluate the potency for large-scale antivenom production.

We foresee many difficulties in this process. First, because supplemental details of current RBS antivenom production were not obtained, we have had to refer to a method described over 60 years ago [5]. Second, not many horses were immunized due to the limited grant fund, raising the possibility that we will be unable to obtain enough antivenom, especially if the horses die. Third, because this is the first time we have attempted to produce RBS antivenom, unexpected problems may occur.

In conclusion, although the domestic production of RBS antivenom has started, great caution is required as we move forward with this plan.
